# The Calcilytic Drug Calhex-231 Ameliorates Vascular Hyporesponsiveness in Traumatic Hemorrhagic Shock by Inhibiting Oxidative Stress and miR-208a-Mediated Mitochondrial Fission

**DOI:** 10.1155/2020/4132785

**Published:** 2020-12-03

**Authors:** Yan Lei, Xiaoyong Peng, Yi Hu, Mingying Xue, Tao Li, Liangming Liu, Guangming Yang

**Affiliations:** ^1^Department of Combat Casualty Care Training, Medical Service Training Base, Army Medical University (Original Third Military Medical University), Chongqing 400038, China; ^2^State Key Laboratory of Trauma, Burns and Combined Injury; Department of Shock and Transfusion, Research Institute of Surgery, Daping Hospital, Army Medical University, Chongqing 400042, China; ^3^Department of Anesthesiology, Daping Hospital, Army Medical University, Chongqing 400042, China

## Abstract

**Background:**

The calcium-sensing receptor (CaSR) plays a fundamental role in extracellular calcium homeostasis in humans. Surprisingly, CaSR is also expressed in nonhomeostatic tissues and is involved in regulating diverse cellular functions. The objective of this study was to determine if Calhex-231 (Cal), a negative modulator of CaSR, may be beneficial in the treatment of traumatic hemorrhagic shock (THS) by improving cardiovascular function and investigated the mechanisms.

**Methods:**

Rats that had been subjected to THS and hypoxia-treated vascular smooth muscle cells (VSMCs) were used in this study. The effects of Cal on cardiovascular function, animal survival, hemodynamics, and vital organ function in THS rats and the relationship to oxidative stress, mitochondrial fusion-fission, and microRNA (miR-208a) were investigated.

**Results:**

Cal significantly improved hemodynamics, elevated blood pressure, increased vital organ blood perfusion and local oxygen supply, and markedly improved the survival outcomes of THS rats. Furthermore, Cal significantly improved vascular reactivity after THS *in vivo and in vitro*. Cal also restored the THS-induced decrease in myosin light chain (MLC) phosphorylation (the key element for VSMC contraction). Inhibition of MLC phosphorylation antagonized the Cal-induced restoration of vascular reactivity following THS. Cal suppressed oxidative stress in THS rats and hypoxic-VSMCs. Meanwhile, THS induced expression of mitochondrial fission proteins Drp1 and Fis1 and decreased expression of mitochondrial fusion protein Mfn1 in vascular tissues. Cal reduced expression of Drp1 and Fis1. In hypoxic-VSMCs, Cal inhibited mitochondrial fragmentation and preserved mitochondrial morphology. In addition, miR-208a mimic decreased Fis1 expression, and miR-208a inhibitor prevented Cal-induced Fis1 downregulation in hypoxic-VSMCs.

**Conclusion:**

Calhex-231 exhibits outstanding potential for effective therapy of traumatic hemorrhagic shock, and the beneficial effects result from its protection of vascular function via inhibition of oxidative stress and miR-208a-mediated mitochondrial fission.

## 1. Introduction

Trauma is the leading cause of death for people under 44 years of age, and about 40% of trauma-related mortality is attributed to hemorrhage and its sequelae [1, 2]. Despite the development of new technologies and therapeutic methods in recent years, the management of trauma/hemorrhage (T/H) patients remains a challenge. Cardiovascular dysfunction, such as vascular hyporesponsiveness, is a well-documented phenomenon and a major cause of death in T/H patients [3–5]. In order to develop more effective treatments, it is necessary to investigate the underlying mechanisms of vascular hyporesponsiveness in trauma and hemorrhagic shock.

The calcium-sensing receptor (CaSR) plays a fundamental role in extracellular calcium homeostasis in humans [6, 7]. Surprisingly, CaSR is also expressed outside of the parathyroid gland and kidney in nonhomeostatic neural and cardiovascular tissues. In the cardiovascular system, functional CaSR is present in cardiomyocytes, perivascular nerves, vascular endothelial cells (VECs), and vascular smooth muscle cells (VSMCs) [8]. Some studies have shown that CaSR plays an important role in the regulation of vascular tone and blood pressure [7–10]. However, the precise functional mechanism of CaSR in the cardiovascular system has not yet been fully clarified.

CaSR can be activated by many kinds of ligands in addition to extracellular calcium (Ca_o_^2+^), the prototypical activator of CaSR. A series of type II allosteric modulators of CaSR have been developed. Positive modulators of CaSR, such as Cinacalcet and Calindol, are named calcimimetics, while negative modulators of CaSR, such as NPS-2143 and Calhex-231, are named calcilytics [11–13]. Recent studies suggest that CaSR modulators may have therapeutic potential in the treatment of cardiovascular disease [14, 15]. However, it is unknown whether CaSR is involved in T/H-induced cardiovascular dysfunction, or if CaSR modulators might exert protective effects on cardiovascular function during traumatic shock.

Oxidative stress and mitochondrial dysfunction play critical roles in the pathogenesis of many cardiovascular diseases. An increased understanding of the tight link between oxidative stress and mitochondria offers tantalizing prospects for the treatment or prevention of these diseases [16–18]. Mitochondrial function is closely linked to the balance between the opposing processes of mitochondrial fusion, controlled by mitofusin 1 (Mfn1) and mitofusin 2 (Mfn 2), and mitochondrial fission, controlled by dynamin-related protein 1 (Drp1) and fission 1 (Fis1) [19, 20]. We previously showed that oxidative stress and mitochondrial dysfunction are both involved in the pathogenesis of vascular hyporesponsiveness following shock. The oxidative damage may be due to an impairment of mitochondrial permeability [5], which is closely related to the status of mitochondrial fusion-fission. In addition, our recent data showed that microRNAs (miRs) play important roles in the regulation of vascular function, and inhibiting CaSR can upregulate the level of miR-208a after shock (unpublished observations). miRs have emerged as critical regulators in mitochondrial fusion and division. However, little is known about whether oxidative stress, mitochondrial fusion-fission, and miRs are involved in the vascular effects of CaSR under shock states.

Based on the literature and our previous findings, we hypothesized that CaSR and its modulators play important roles in cardiovascular function, possibly via a mechanism that is related to oxidative stress and mitochondrial dynamic processes. In this study, we tested whether the calcilytic Calhex-231 improves cardiovascular function when used to treat traumatic hemorrhagic shock (THS). We also investigated the relationship with oxidative stress, mitochondrial fusion-fission, and miR-208a, using THS-induced rats and hypoxia-treated VSMCs.

## 2. Materials and Methods

### 2.1. Ethics

The investigation conformed to “Practical guidelines for rigor and reproducibility in preclinical and clinical studies on cardioprotection” [21] and “Guide for the Care and Use of Laboratory Animals” (Eighth Edition, 2011, Washington, D.C., National Academies Press, USA). The study protocol was approved by the Laboratory Animal Welfare and Ethics Committee of the Third Military Medical University.

### 2.2. Experimental Interventions on Rats

**Figure 1:**
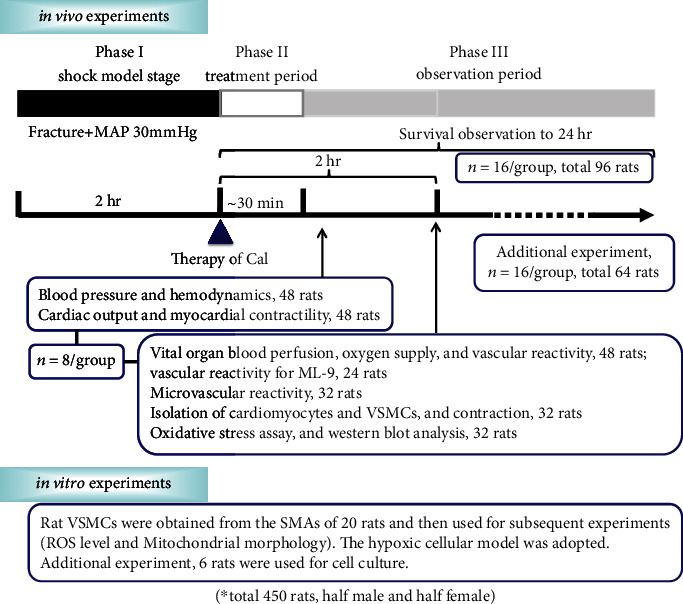
Schematic representation of the experimental protocol *in vivo and in vitro*.

Four hundred and fifty Sprague–Dawley (SD) rats (half male and half female) were used in the current study. A rat model of traumatic hemorrhagic shock was induced as previously described [5]. For *in vivo* experiments, rats were randomly divided into six groups: normal control (sham-operated), shock control, shock+lactated Ringer's (LR) solution, and shock+LR+Calhex-231 at 0.1, 1, or 5 mg/kg. Protocols for animal models, groups, drug administration, and assays are described in *Methods in the Supplemental Material*. In addition, we also observed the effect of LR in normal rats and the effect of Calhex-231 alone (without LR resuscitation) in THS rats (detailed in *Supplemental Figures in the Supplemental Material*). A schematic diagram outlining the experimental protocol is presented in Figure 1.

### 2.3. Animal Survival

After resuscitation and group-specific treatments, animal survival was observed for 24 hours.

### 2.4. Blood Pressure and Hemodynamics

Mean arterial pressure (MAP), the pressor response of norepinephrine (NE), and hemodynamic parameters (LVSP (left intraventricular systolic pressure) and ±dp/dt_max_ (maximal rate of change in left intraventricular pressure)) were measured before hemorrhage (baseline), at the end of the shock period, and at 1 and 2 hours after resuscitation.

### 2.5. Cardiac Output and Myocardial Contractility

Cardiac output (CO) was measured at baseline, at the end of the shock period, and at 1 and 2 hr after resuscitation. Subsequently, the papillary muscles of rats were isolated, and the contractility was measured.

### 2.6. Vital Organ Blood Perfusion, Local Oxygen Supply, and Vascular Reactivity of Isolated Mesenteric Vessels

Two hours after resuscitation, blood perfusion and oxygen saturation of the liver and kidney were assessed. Then, the superior mesenteric arteries (SMAs) of rats were isolated, and the vascular reactivity to NE was measured.

### 2.7. Microvascular Reactivity of Mesenteric Microvessels *In Vivo*

Two hours after resuscitation, the mesenteric microvessels were observed under an inverted intravital microscope, and the contractile response of mesenteric arterioles was assessed by measuring the change in diameter in response to increasing doses of NE.

### 2.8. Isolation of Cardiomyocytes and VSMCs and Contraction in Isolated Myocytes

Two hours after resuscitation, the hearts and mesenteric arteries were rapidly removed. Dissociated cardiomyocytes were isolated by a collagenase perfusion method, and cardiomyocyte contraction was observed using an IonOptix edge-detection system [22]. Dissociated smooth muscle cells were isolated using an enzymatic method, and the contractile response to NE was determined using a Leica confocal system.

### 2.9. Oxidative Stress Assay, Preparation of Tissue Lysates, and Western Blot Analysis

Two hours after resuscitation, blood samples and SMA tissues were collected. Serum levels for four oxidative stress biomarkers, including malondialdehyde (MDA), superoxide dismutase (SOD), catalase (CAT), and glutathione (GSH), were detected with commercial assay kits. SMA tissue protein extracts were prepared, and western blot analysis was performed as described previously [3].

### 2.10. Primary Culture of VSMCs and Hypoxic Treatment

Rat VSMCs were obtained from the SMAs of SD rats using an explant technique, and the hypoxic treatment was performed as previously described [3].

### 2.11. ROS Level and Mitochondrial Morphology in VSMCs

The cultured VSMCs were divided into three groups: normal control, hypoxia 2 hr, and hypoxia+Cal (10 *μ*mol/L, 30 min). Reactive oxygen species (ROS) levels in VSMCs were measured using the 2′,7′-dichlorofluorescin diacetate (DCF-DA) method [5]. The morphology of rat VSMC mitochondria stained with MitoTracker deep red was detected using a Leica confocal microscope.

### 2.12. Oligonucleotide Transfection

Synthetic miR-208a mimic, miRNA negative control, miR-208a inhibitor, and miRNA inhibitor negative control were purchased from RiBoBio (Guangzhou, China). VSMCs were transfected with miR-208a mimic and its negative control (100 nmol/L) or miR-208a inhibitor and correlated negative control oligonucleotides (200 nmol/L) using Lipofectamine 2000 (Invitrogen, Carlsbad, CA, USA) according to the manufacturer's protocol.

### 2.13. Isolation of Mitochondria from VSMCs and Western Blot Analysis

The isolation of mitochondria from VSMCs was obtained with the Mitochondria Isolation Kit (Sigma). Mitochondrial extracts were prepared, and western blot analysis was performed.

### 2.14. Statistical Analysis

Data are presented as means ± standard error (SEM). Animal survival was analyzed using the Kaplan-Meier survival curve. The SPSS software was used to compare experiments using one or two-factor analysis of variance analyses. *P* < 0.05 was considered significant.

## 3. Results

### 3.1. Effect of Calhex-231 on Survival Time and 24 hr Survival Rate in Rats with THS

**Figure 2:**
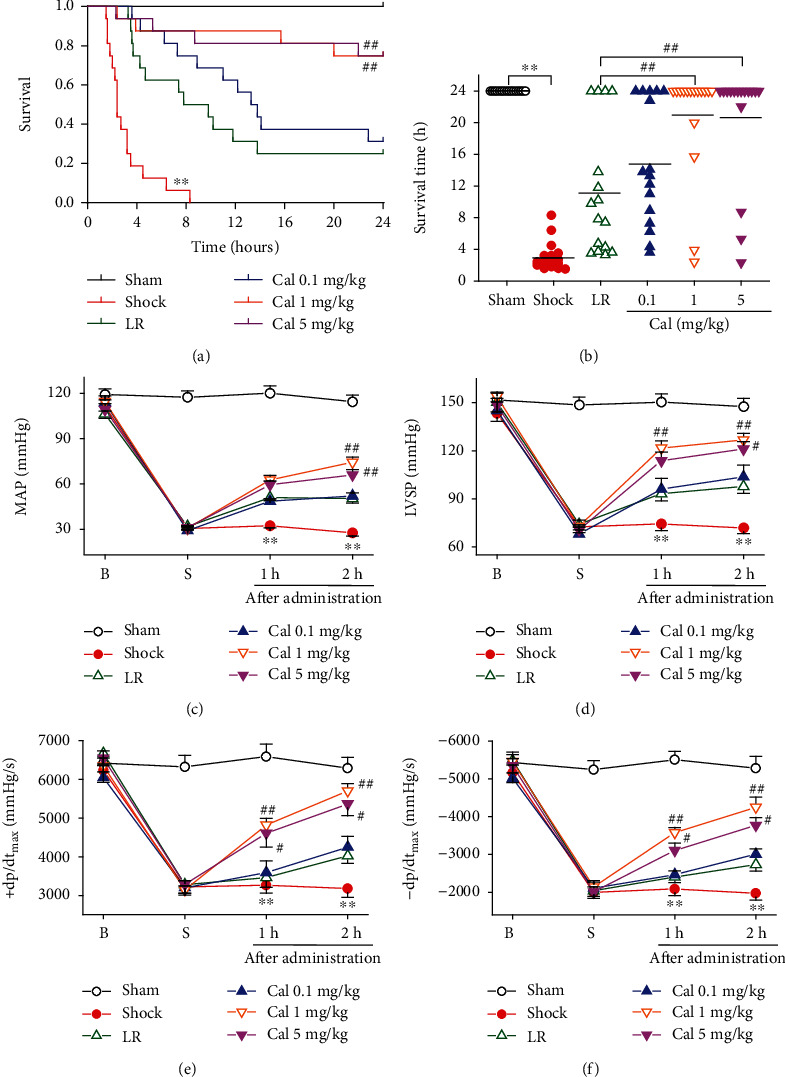
Effect of Calhex-231 on survival and hemodynamic parameters in rats with THS: (a, b) Kaplan-Meier survival curve and survival time (*n* = 16/group); (c–f) hemodynamics, including mean arterial pressure (MAP), left intraventricular systolic pressure (LVSP), and maximal change rate in left intraventricular pressure (±dp/dt_max_) (*n* = 8/group). ^∗∗^*P* < 0.01 compared with the sham-operated group; #*P* < 0.05, ##*P* < 0.01 compared with the LR group. Sham: sham-operated; LR: lactated Ringer's solution; Cal: Calhex-231; B: baseline; S: shock.

Administration of 5 or 1 mg/kg Cal significantly increased survival time and the 24 hr survival rate of rats suffering traumatic hemorrhagic shock (*P* < 0.01). The survival time and 24 hr survival rate in these two groups were statistically indistinguishable. Rats in the 0.1 mg/kg Cal group had slightly increased survival time and 24 hr survival rate compared to rats in the LR only group, but the difference was not statistically significant (Figures 2(a) and 2(b)). In addition, LR infusion did not alter the survival status in normal rats (Supplemental Fig. S1A). When compared with Cal with LR resuscitation, Cal (1 mg/kg) without LR infusion did not improve the survival of THS rats (Supplemental Fig. S2A).

### 3.2. Effect of Calhex-231 on Blood Pressure and Hemodynamics in Rats with THS

In all groups, MAP, LVSP, and ±dp/dt_max_ decreased significantly after shock. Administration of 5 or 1 mg/kg Cal resulted in significantly increased values at 1 and 2 hr postadministration, compared to rats in the LR only group (*P* < 0.05or 0.01). Rats treated with 1 mg/kg Cal demonstrated the greatest recovery (Figures 2(c)–2(f)). In addition, LR infusion induced short-term and slightly increase of blood pressor in normal rats (Supplemental Fig. S1B). Cal (1 mg/kg) without LR infusion did not restore the decreased MAP after shock (Supplemental Fig. S2B).

### 3.3. Effect of Calhex-231 on Blood Perfusion and Oxygen Saturation of the Liver and Kidney in Rats with THS

**Figure 3:**
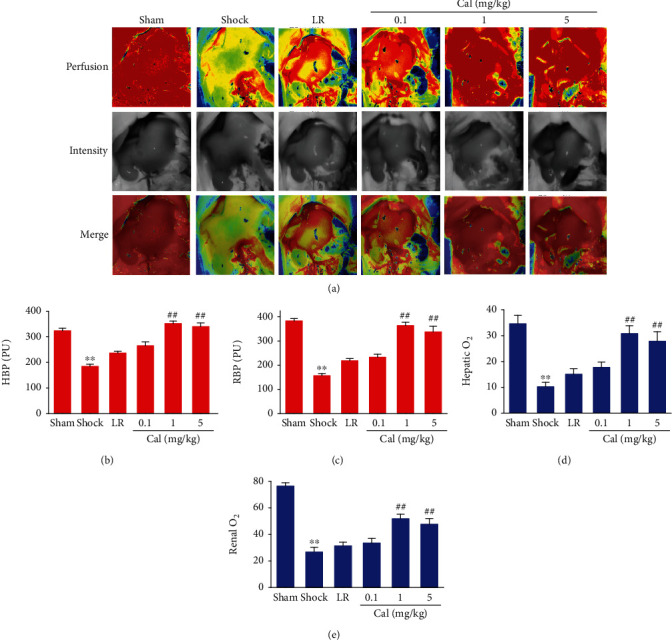
Effect of Calhex-231 on blood perfusion and oxygen saturation of the liver and kidney in rats with THS: (a) representative LASCA images of blood perfusion in the liver and kidney; (b, c) graphical representation and statistical analysis of hepatic (HBP) and renal blood perfusion (RBP); (d, e) oxygen saturation of the liver and kidney. ^∗∗^*P* < 0.01 compared with the sham-operated group; ##*P* < 0.01 compared with the LR group. Sham: sham-operated; LR: lactated Ringer's solution; Cal: Calhex-231. *n* = 8/group.

A significant decrease in blood perfusion in the liver and kidney was observed after shock. Administration of 5 or 1 mg/kg Cal resulted in significantly increased perfusion in both the liver and kidney, compared to rats in the LR group (*P* < 0.01) (Figures 3(a)–3(c)). Not unexpectedly, the oxygen saturation results mirrored the blood perfusion results (Figures 3(d) and 3(e)).

### 3.4. Effect of Calhex-231 on Cardiac Function in Rats with THS

**Figure 4:**
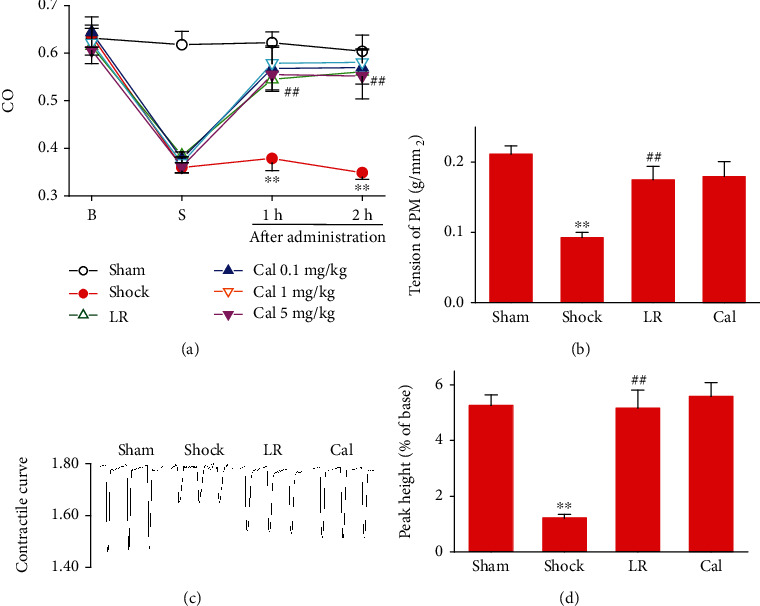
Effect of Calhex-231 on cardiac function in rats with THS: (a) cardiac output (CO); (b) contractility of isolated ventricular papillary muscle (PM); (c) representative curves of cardiomyocyte contraction; (d) contractility of single cardiomyocytes. ^∗∗^*P* < 0.01 compared with the sham-operated group; ##*P* < 0.01 compared with the LR group. Sham: sham-operated; LR: lactated Ringer's solution; Cal: Calhex-231; B: baseline; S: shock. *n* = 8/group.

Cardiac function was evaluated *in vivo* by measuring cardiac output and *in vitro* by measuring the contractility of isolated ventricular papillary muscle and single cardiomyocytes. All the indicators for heart function decreased after shock, and LR infusion significantly increased these indicators. There were no significant differences in the Cal group compared with the LR group (Figures 4(a)–4(d)).

### 3.5. Effect of Calhex-231 on Vascular Function in Rats with THS

**Figure 5:**
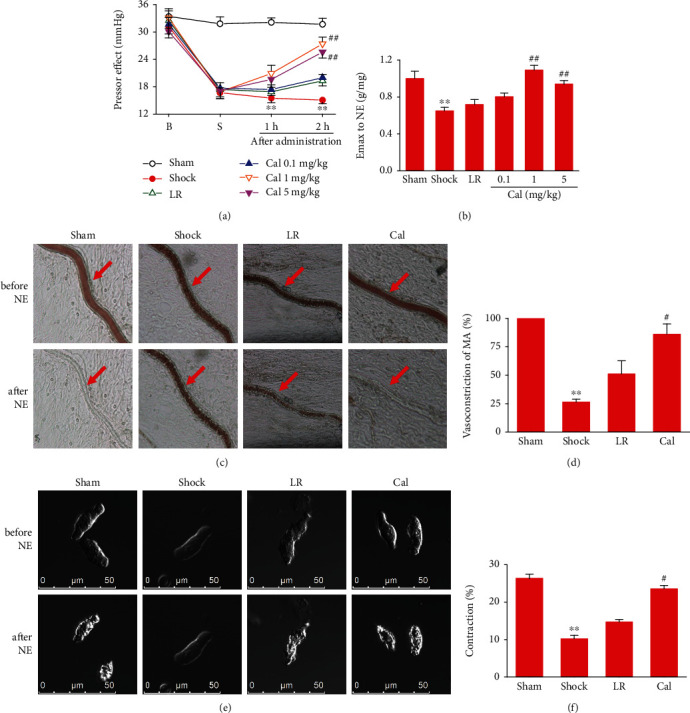
Effect of Calhex-231 on vascular function in rats with THS: (a) pressor effect of norepinephrine (NE); (b) contractile response of superior mesenteric arteries (SMA) to NE; (c, d) contractile response of mesenteric arterioles (MA) to NE; (e, f) contractility of single VSMCs. ^∗∗^*P* < 0.01 compared with the sham-operated group; #*P* < 0.05, ##*P* < 0.01 compared with the LR group. Sham: sham-operated; LR: lactated Ringer's solution; Cal: Calhex-231; B: baseline; S: shock. *n* = 8/group.

To determine the effects of Cal on vascular function (vascular reactivity) following THS, we investigated pressor effect of NE and the contractile response of superior mesenteric arteries (SMA), mesenteric arterioles, and isolated VSMCs to NE *in vivo and in vitro*. The pressor effect of NE (reflecting vascular reactivity *in vivo*) in THS rats decreased significantly, consistent with our previous report [5]. LR infusion slightly improved the pressor response to NE. Cal treatments significantly increased the pressor effect of NE as compared with the LR group (*P* < 0.01) (Figure 5(a)). Similarly, the constriction reactivity of isolated SMAs from THS rats was significantly reduced, and LR improved slightly. Cal markedly increased the constriction of SMAs from THS rats (Figure 5(b)). Similar results were obtained in the mesenteric arterioles and isolated VSMCs (Figures 5(c)–5(f)).

### 3.6. Effect of Calhex-231 on Oxidative Stress

**Figure 6:**
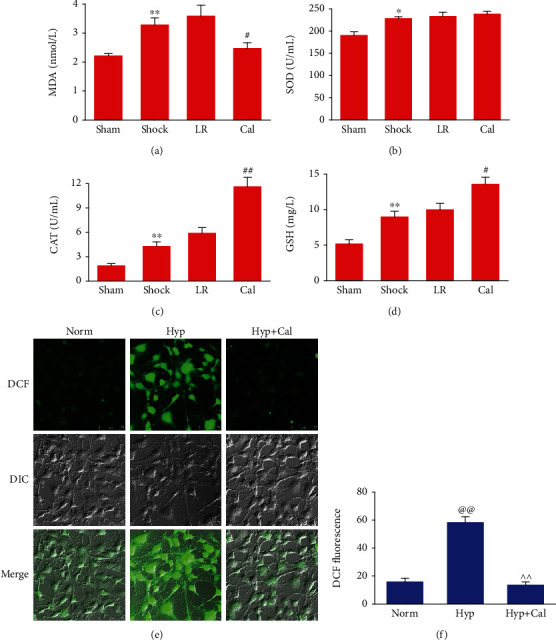
Effect of Calhex-231 on oxidative stress in vivo and in vitro: (a–d) levels of malondialdehyde (MDA), superoxide dismutase (SOD), catalase (CAT), and glutathione (GSH) in blood samples (*n* = 8/group); (e) images acquired by confocal microscopy showing intracellular ROS levels in VSMCs; (f) quantitative data of the mean intensity of 2′,7′-dichlorofluorescin (DCF, a probe of ROS) fluorescence (*n* = 30 cells/group). ^∗^*P* < 0.05, ^∗∗^*P* < 0.01 compared with the sham-operated group; #*P* < 0.05, ##*P* < 0.01 compared with the LR group; @@*P* < 0.01 compared with the normal group; ^^*P* < 0.01 compared with the hypoxia group. Sham: sham-operated; LR: lactated Ringer's solution; Cal: Calhex-231; Norm: normal; Hyp: hypoxia.

MDA (the sensitive indicator of oxidative stress) levels in blood samples from THS rats increased significantly. In parallel, levels of the protective antioxidant enzymes SOD, CAT, and GSH also increased. Levels of these biomarkers did not change significantly after resuscitation with LR alone. Administration of Cal significantly decreased MDA level and further increased CAT and GSH levels, as compared with the LR group (*P* < 0.05 or 0.01) (Figures 6(a)–6(d)). Under hypoxic conditions, intracellular ROS levels in VSMCs increased significantly. Treatment with Cal markedly reduced ROS levels in hypoxic-VSMCs (Figures 6(e) and 6(f)).

### 3.7. Role of MLC Phosphorylation in Cal-Mediated Effects on Vascular Reactivity after THS

**Figure 7:**
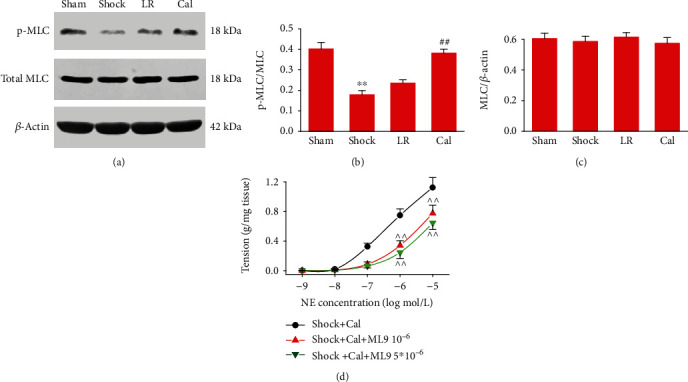
Role of MLC phosphorylation in Cal-mediated effects on vascular reactivity after THS: (a) representative immunoblots showing the expression and phosphorylation of MLC; (b) ratio of phosphorylated MLC/total MLC based on optical density of immunoblot bands; (c) ratio of total MLC/*β*-actin based on optical density of immunoblot bands. Immunoblot analyses were repeated three times. (d) Contractile response of superior mesenteric arteries to norepinephrine (NE) (*n* = 8/group). ^∗∗^*P* < 0.01 compared with the sham-operated group; ##*P* < 0.01 compared with the LR group; ^^*P* < 0.01 compared with the shock+Cal group. Sham: sham-operated; LR: lactated Ringer's solution; Cal: Calhex-231.

Immunoblot analysis revealed that the phosphorylation of MLC in blood vessels decreased significantly after shock, and LR infusion had no significant influence on MLC phosphorylation. Cal treatment significantly increased MLC phosphorylation, while MLC protein expression did not change (Figures 7(a)–7(c)). Furthermore, two doses of ML-9 (a selective MLCK inhibitor that inhibits phosphorylation of MLC) antagonized the Cal-induced restoration of vascular reactivity of SMA after shock (*P* < 0.01) (Figure 7(d)).

### 3.8. Effect of Calhex-231 on Mitochondrial Fission/Fusion and Mitochondrial Morphology following THS

**Figure 8:**
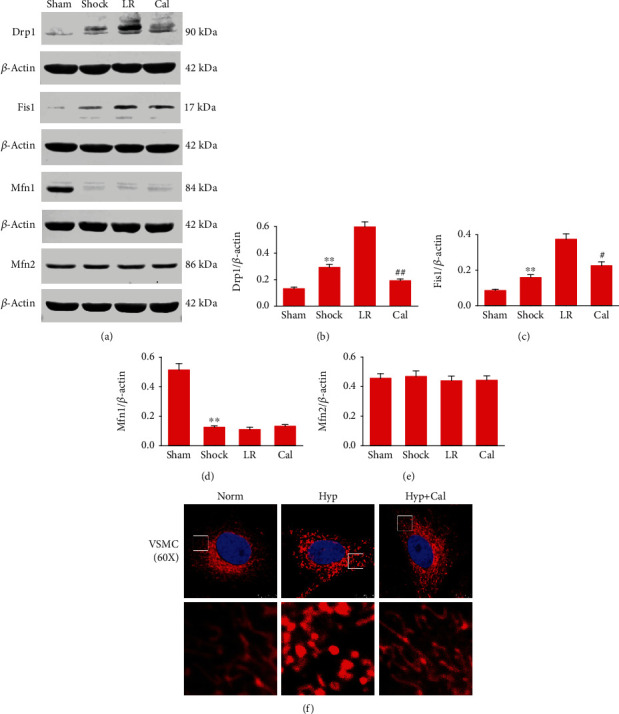
Effect of Calhex-231 on mitochondrial fission/fusion and mitochondrial morphology following THS: (a) representative immunoblots showing expression of Drp1, Fis1, Mfn1, and Mfn2; (b–e) protein levels normalized to *β*-actin based on optical density of immunoblot bands. Immunoblot analyses were repeated three times. (f) Representative confocal images showing morphology of rat VSMC mitochondria stained with MitoTracker deep red. Red, MitoTracker fluorescence; blue, DAPI fluorescence. The boxed areas in the top images are shown at higher magnification in the bottom images. Experiments were repeated three times. ^∗∗^*P* < 0.01 compared with the sham-operated group; #*P* < 0.05, ##*P* < 0.01 compared with the LR group. Sham: sham-operated; LR: lactated Ringer's solution; Cal: Calhex-231; Norm: normal; Hyp: hypoxia.

The expression of Drp1 and Fis1, two important mitochondrial fission proteins, increased significantly in blood vessels from THS rats. Cal treatment significantly reduced the Drp1 and Fis1 expression (Figures 8(a)–8(c)). Expression of the fusion protein Mfn1 decreased significantly after shock, while Cal had no significant influence on Mfn1 levels under shock conditions (Figures 8(a) and 8(d)). Expression of Mfn2, another fusion protein, did not change significantly after shock and/or Cal treatment (Figures 8(a) and 8(e)).

Confocal microscopy showed that hypoxia caused significant changes in mitochondrial morphology in rat VSMCs. Most mitochondria in normal cells were tubular, branched, and displayed a typical networked morphology. However, exposure to hypoxia disrupted the elongated networked structure, and mitochondria became shorter and fragmented. Treatment with Cal significantly reduced the hypoxia-induced mitochondrial fragmentation in VSMCs, as measured by the incidence of fragmented mitochondria and the formation of elongated networks (Figure 8(f)).

### 3.9. Role of miR-208a in Cal-Regulation of Mitochondrial Fission Proteins

**Figure 9:**
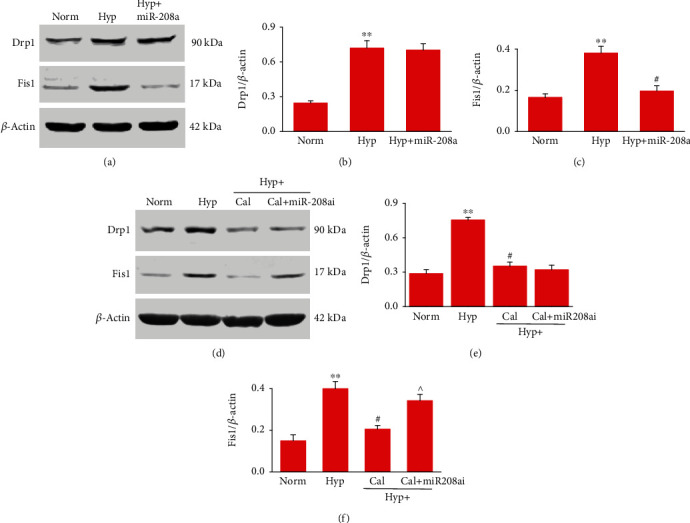
Role of miR-208a in Cal-regulation of mitochondrial fission proteins in hypoxic-VSMCs: (a) effect of miR-208a on the protein expression of Drp1 and Fis1 in hypoxic-VSMCs; (d) role of miR-208a in the regulation of the Drp1 and Fis1 expression by Cal. Representative immunoblots showing expression of Drp1 and Fis1. (b, c, e, and f) Protein levels normalized to *β*-actin based on optical density of immunoblot bands. Immunoblot analyses were repeated three times. ^∗∗^*P* < 0.01 compared with the normal group; #*P* < 0.05 compared with the hypoxia group; ^*P* < 0.05 compared with the hypoxia+Cal group. Norm: normal; Hyp: hypoxia; miR-208a: miR-208a mimic; miR-208ai: miR-208a inhibitor; Cal: Calhex-231.

We then investigated the potential mechanisms that are involved in Cal-regulating mitochondrial fission. Our recent studies showed that Cal increased the level of miR-208a and thereby enhanced the contractile response of SMAs after hemorrhagic shock (unpublished observations). Here, we show that in hypoxic-VSMCs, the expression of Drp1 and Fis1 also increased, and in the presence of miR-208a mimic, the expression of Fis1, not Drp1, was decreased (Figures 9(a)–9(c)). Moreover, treatment with the miR-208a inhibitor abolished the Cal-induced decrease in the Fis1 expression, whereas did not affect Drp1 levels following Cal (Figures 9(d)–9(f)). Similar changes were also observed in isolated mitochondria from VSMCs (Supplemental Fig. S3). These results suggest that Cal upregulated the level of miR-208a and subsequently inhibited mitochondrial fission protein Fis1 in hypoxic-VSMCs.

## 4. Discussion

Our results demonstrate that Calhex-231 (Cal), a specific inhibitor of CaSR, has a mitigating effect on traumatic hemorrhagic shock by improving vascular hyporesponsiveness and reducing mitochondrial dysfunction. First, application of Cal significantly improved survival outcomes in THS rats. Second, Cal exerts its protective effect by regulating vascular contraction and concomitant MLC phosphorylation in smooth muscle, thereby recovering vascular hyporesponsiveness after THS. Finally, the vascular function protection conferred by Cal may be attributed to attenuation of oxidative stress and reversal of damage to mitochondrial morphology and function, and miR-208a is involved in Cal-regulating mitochondrial fission. These findings show the important role played by CaSR in the regulation of vascular reactivity after THS, and the potential offered by its allosteric modulator Calhex-231 for the treatment of critical illness.

The functional relevance of CaSR on blood pressure is the subject of controversy. Several studies show that the calcimimetic R-568 induces a sustained reduction in blood pressure in uremic and spontaneously hypertensive rats but not in normal rats [23]. The calcilytic NPS-2143 increases blood pressure in normotensive rats [24]. However, Fryer et al. [25] reported that Cinacalcet (the only calcimimetic approved for clinical use) produces an acute increase in blood pressure in both uremic and normal rats. In *in vitro* experiments, NPS-2143 inhibits vascular contraction induced by vasoconstrictors in rat mesenteric arteries exposed to hypoxia/reoxygenation [26]. These conflicting results may be related to the fact that CaSR is expressed in a diverse range of tissues or that the experiments were conducted in different pathophysiologic states or used different methods for drug administration.

In this study, we first evaluated the therapeutic effects of intravenous infusion of Calhex-231 in rats subjected to THS. Our results show that compared with resuscitation by LR alone, Cal provides therapeutic benefit in traumatic hemorrhagic shock. Next, to investigate whether the cardiovascular actions of Cal contribute to its protective effects against THS, we observed the effects of Cal on cardiac function and vascular function *in vivo* and *in vitro*. Our data suggested that Cal elicits the beneficial effect by improving vascular function, not heart function. Subsequent experiment investigated the relationship between CaSR and vascular reactivity, and the data show that Cal restores the THS-induced decrease in MLC phosphorylation, which is the principal mechanism responsible for VSMC contraction and vascular reactivity. These results demonstrate that the calcilytic drug Calhex-231 has a beneficial effect on THS by enhancing VSMC contraction and protecting vascular function.

To explore the mechanisms by which Calhex-231 improves vascular hyporesponsiveness following THS, we studied the relationships between the vascular action of Cal, oxidative stress, and mitochondrial dysfunction. In the present study, THS resulted in increased oxidative stress, confirming our previous observations [5]. Treatment with Cal suppressed oxidative stress in THS rats and markedly reduced ROS levels in hypoxic-VSMCs, suggesting the calcilytic compound Calhex-231 has antioxidant activity. We also found that hypoxia damages mitochondrial morphology and results in significant mitochondrial fragmentation in VSMCs. However, Cal treatment inhibits this fragmentation and preserves mitochondrial morphology. Mitochondria are highly dynamic, and their morphological changes correlate highly with function and cell fate. Mitochondrial morphology is regulated by the processes of fusion and fission. The morphological regulatory factors include the GTPases Drp1 and Fis1, which mediate mitochondrial fission, and the homologous GTPases Mfn1 and Mfn2, which mediate mitochondrial fusion [27]. We therefore explored potential interactions between the vascular effects of Cal and mitochondrial fusion/fission proteins. Our data suggest that the protection conferred by Cal on mitochondrial morphology is due to inhibitory effects on the mitochondrial fission proteins Drp1and Fis1.

A further finding presented here is that miR-208a is involved in Cal-regulating mitochondrial fission. MicroRNAs have been known to play critical roles in the regulation of gene expression [28]. Several miRs are reported to participate in the regulation of mitochondrial dynamics, such as miR-668 and miR-181a [29, 30]. In our present study, transfection of the miR-208a mimic into VSMCs decreased the expression of Fis1, and inhibition of miR-208a eliminated the inhibitory effects of Cal on the Fis1 expression under hypoxic conditions. Taken together, with our recent data that Cal induced upregulation of miR-208a after THS (unpublished observations), it is suggested that Cal inhibits mitochondrial fission protein Fis1 expression by elevating the level of miR-208a (Supplemental Fig. S4). In light of our previous studies and results reported by others, we speculate that Cal primarily protects vascular function by counteracting oxidative stress and miR-208a-mediated mitochondrial fission. The precise mechanism by which Cal confers these benefits needs further investigation.

This study raises several important questions. Since CaSR is widely expressed in many tissues and organs, does it have additional antishock functions beyond those that result in vascular protection? What mechanisms are responsible for the crosstalk that occurs between oxidative stress, miRs, and mitochondrial dysfunction? Whether or not the role of CaSR under shock conditions is affected by gender? Whether and how nitric oxide (the important molecule in the pathogenesis of shock) participates in the effects of CaSR after THS? Can these mechanisms be exploited to develop novel treatment strategies for traumatic hemorrhagic shock?

## 5. Conclusions

In summary, our study demonstrates that the calcilytic drug Calhex-231 exhibits great potential as an effective therapeutic agent in the treatment of traumatic hemorrhagic shock. Cal treatment improves hemodynamic parameters and vital organ blood perfusion and markedly prolongs survival in THS rats. The beneficial effects of Cal result from its ability to protect vascular function via inhibition of oxidative stress and miR-208a-mediated mitochondrial fission.

## Data Availability

The data used to support the findings in this paper are available from the corresponding author upon request.

## Supplementary Materials

Supplementary Materials Supplemental Figure S1. Effect of LR on animal survival and blood pressor in normal rats (n=16/group). A: survival time; B: mean arterial pressure (MAP). ∗*P* <0.05, ∗∗*P* <0.01 compared with the normal control group. LR, lactated Ringer's solution; B, baseline; S, end of shock, Supplemental Figure S2. Effect of Cal with or without LR resuscitation on animal survival and blood pressor after traumatic hemorrhagic shock. (n=16/group). A: survival time; B: mean arterial pressure (MAP). ∗*P* <0.05, ∗∗*P* <0.01 compared with the Cal with LR group.; LR, lactated Ringer's solution; Cal, Calhex-231; B, baseline; S, end of shock, Supplemental Figure S3. Role of miR-208a in Cal regulating expression of Drp1 and Fis1 in isolated mitochondria from VSMCs. A: effect of miR-208a on the protein expression of Drp1 and Fis1 in in isolated mitochondria from VSMCs. B: role of miR-208a in the regulation of Drp1 and Fis1 expression by Cal in isolated mitochondria from VSMCs. Norm, normal; Hyp, hypoxia; miR-208a, miR-208a mimic; miR-208ai, miR-208a inhibitor; Cal, Calhex-231. Supplemental Figure S4. Schematic diagram showing the possible signaling mechanisms of Calhex-231's effects under shock states.
