# Supplementary material for: The Calcilytic Drug Calhex-231 Ameliorates Vascular Hyporesponsiveness in Traumatic Hemorrhagic Shock by Inhibiting Oxidative Stress and miR-208a-Mediated Mitochondrial Fission
Source: Oxid Med Cell Longev. 2020 Dec 3;2020:4132785. doi: 10.1155/2020/4132785 (PMC7732383; doi:10.1155/2020/4132785)
Supplement: Supplementary Materials — Supplemental Figure S1. Effect of LR on animal survival and blood pressor in normal rats (n=16/group). A: survival time; B: mean arterial pressure (MAP). ∗P <0.05, ∗∗P <0.01 compared with the normal control group. LR, lactated Ringer's solution; B, baseline; S, end of shock, Supplemental Figure S2. Effect of Cal with or without LR resuscitation on animal survival and blood pressor after traumatic hemorrhagic shock. (n=16/group). A: survival time; B: mean arterial pressure (MAP). ∗P <0.05, ∗∗P <0.01 compared with the Cal with LR group.; LR, lactated Ringer's solution; Cal, Calhex-231; B, baseline; S, end of shock, Supplemental Figure S3. Role of miR-208a in Cal regulating expression of Drp1 and Fis1 in isolated mitochondria from VSMCs. A: effect of miR-208a on the protein expression of Drp1 and Fis1 in in isolated mitochondria from VSMCs. B: role of miR-208a in the regulation of Drp1 and Fis1 expression by Cal in isolated mitochondria from VSMCs. Norm, normal; Hyp, hypoxia; miR-208a, miR-208a mimic; miR-208ai, miR-208a inhibitor; Cal, Calhex-231. Supplemental Figure S4. Schematic diagram showing the possible signaling mechanisms of Calhex-231's effects under shock states. [file 4132785.f1.doc]

**Supplemental Material**

**1. Supplemental Methods**

**2. Supplemental Figure S1-S4 (including Figures and FIGURE LEGENDS)**

**1. Supplemental Methods**

**Ethics**

The study protocol was approved by the Laboratory Animal Welfare and Ethics Committee Of the Third Military Medical University. The present study is a part of the study about calcium-sensing receptor, the approval number in the whole study is 1000 SD rats (showing below), and the used animal number in this present study is 450 rats.


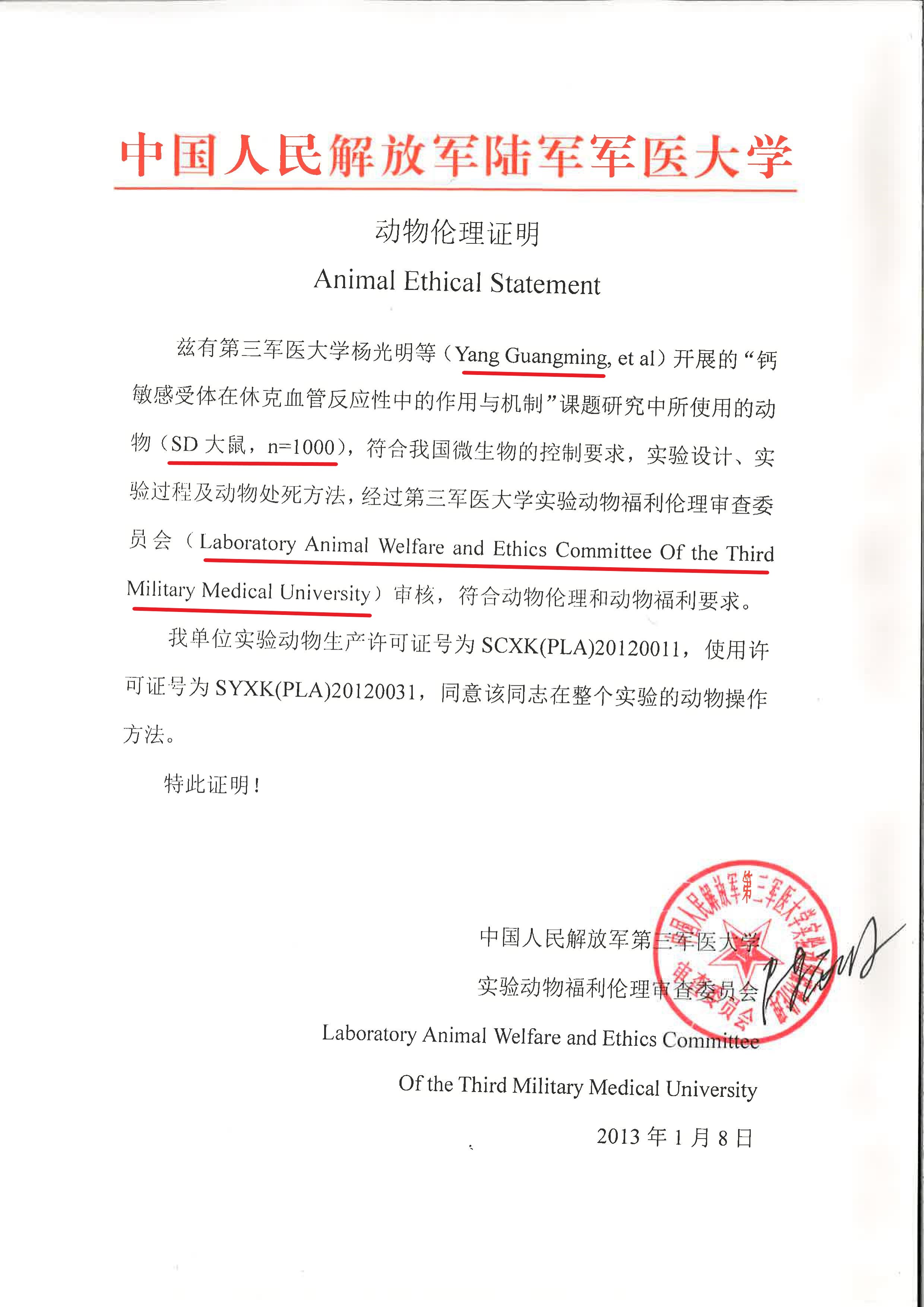


Animal Ethical Statement

**Animals**

SD rats were housed in a containment facility at the animal center at an ambient temperature of 25 °C and a light/dark cycle of 12 hr each. Animals were provided with food and water *ad libitum*. Food was withdrawn 12 hr prior to the experiment, but access to water was maintained.

**Traumatic hemorrhagic shock models and resuscitation**

Rats were anesthetized with sodium pentobarbital (initial dosage, 30 mg/kg ip) and Jingsongling (xylidinothiazole; initial dosage, 0.1 mg/kg im). The heparinized catheters were placed in the right femoral artery and vein. Blood pressure and bleeding were monitored from the femoral artery and drugs were administered through the femoral vein. The right carotid artery and vein were catheterized for monitoring hemodynamic values or cardiac output. Traumatic hemorrhagic shock was induced as previously described. Briefly, a fracture of the left femur was made, and hemorrhage was induced via the right femoral artery catheter until mean arterial pressure (MAP) decreased to 30 mmHg. After maintaining this pressure for 2 hours, traumatic hemorrhagic shock was established and experiments were conducted. At the conclusion of all experiments, animals were euthanatized with a pentobarbital-based euthanasia solution (Sleepaway, 2 ml iv; Fort Dodge Laboratories, Fort Dodge, IA, USA).

For *in vivo* experiments, rats were randomly divided into six groups: normal control (sham-operated), shock control, shock+lactated Ringer’s (LR) solution, and shock+LR+Calhex-231 at 0.1, 1, or 5 mg/kg (Tocris Bioscience, Bristol, UK). When shock had been established, the animals in the three Calhex-231 (Cal) groups received a continuous infusion of 0.1, 1, or 5 mg/kg Cal in LR solution within 30 minutes. The LR group received only lactated Ringer’s solution. In these groups the volume of LR was twice the volume of blood lost. The shock control group did not receive any treatment after shock. The sham-operated rats experienced the same operation but no shock was induced and they received no LR infusion. LR was administered with an infusion pump (Model AS 50, B. Braun Melsungen AG, Germany).

In addition, a supplementary experiment observed the effect of LR on animal survival and blood pressor in normal rats, and the effect of Calhex-231 alone (without LR resuscitation) on the indexes in traumatic hemorrhagic shock rats. Rats were divided four groups: normal control and normal+LR; shock+LR+Calhex-231 1 mg/kg and shock+Calhex-231 1 mg/kg (without LR).

**Animal survival**

Ninety-six rats were randomly divided into the six groups (n=16/group) and subjected to the procedures described above. After resuscitation and group-specific treatments, all catheters were removed and the incisions were closed in two layers with nonabsorbable suture. Rats were returned to their cages and allowed free access to food and water. Postoperative analgesia (xylidinothiazole 0.2 mg/kg) was administered intramuscularly every 6 hr. Rats were observed every 1 hr for 24 hours.

In addition, 64 SD rats for the supplementary experiment were divided four groups (n=16/group) that described above. Blood pressure were measured at baseline, at the end of the shock period, and at 1 and 2 hours after resuscitation. After measurement, the animal survival was observed.

**Blood pressure and hemodynamics**

Forty-eight rats were randomly divided into the six groups (n=8/group) and subjected to the procedures described earlier. Mean arterial pressure (MAP), the pressor response of norepinephrine (NE), and hemodynamic parameters were measured before hemorrhage (baseline), at the end of the shock period, and at 1 and 2 hours after resuscitation. MAP was monitored using the right femoral artery catheter connected to a blood pressure analyzer. The pressor response was represented as the maximum increase in MAP after administration of NE (3 μg/kg bolus intravenous injection), which reflects the *in vivo* systemic vascular reactivity. Hemodynamic parameters, including LVSP (left intraventricular systolic pressure), and ±dp/dt_max_ (maximal rate of change in left intraventricular pressure) were determined with a polygraph physiologic recorder (SP844; AD Instruments, Castle Hill, Australia).

**Cardiac output and myocardial contractility**

Forty-eight rats were used for this experiment, randomly divided into six groups and subjected to the procedures described above. Using a Cardiomax-III Thermodilution Cardiac Output System (Columbus Instruments, Columbus, OH, USA), cardiac output (CO) was measured at baseline, at the end of the shock period, and at 1 and 2 hr after resuscitation. Subsequently, rats were euthanatized and the hearts were immediately isolated and placed in Tyrode’s solution (mmol/L: 137 NaCl, 5.4 KCl, 1.2 MgCl_2_, 10 HEPEs, 1.2 CaCl_2_, 1.2 NaH_2_PO_4_, 10 glucose), continuously infused with 95 % O_2_ /5 % CO_2_. A papillary muscle bundle was dissected from the left ventricle and mounted in an organ bath continuously perfused with oxygenated Tyrode’s solution for 2 hr at 30 ºC. Papillary muscles were electrically stimulated by 10 ms square wave pulses at a rate of 0.2 Hz and a voltage 20 % above threshold. After equilibration for 1 hr at a resting tension of 0.5 g, the muscles were adjusted to optimum length, followed by another 30 min period of equilibration. Contractility of the isolated papillary muscle was analyzed under increasing isoproterenol concentrations (10^-9^ to 10^-4^ mol/L), and the contractile force was recorded with a Power Lab System via a force transducer (AD Instruments, Castle Hill, Australia). Papillary muscle contractility, normalized for muscle cross-sectional area (g/mm^2^), was calculated using the formula: G/π(D/2)^2^ (G: tension; D: diameter).

**Vital organ blood perfusion, local oxygen supply, and vascular reactivity of isolated mesenteric vessels**

Forty-eight rats were divided into six groups (n=8/group) and subjected to the treatments described above. Two hours after resuscitation, blood perfusion of the liver and kidney was assessed by laser speckle contrast analysis (LASCA) using a PeriCam PSI system (PSI-ZR; Perimed, Jarfalla, Sweden). Briefly, the rats were placed in the supine position, sodium pentobarbital and xylidinothiazole anesthesia was maintained, and a laparotomy was performed exposing the liver and kidney. Dynamic blood perfusion was recorded in real-time with a PSI camera, and the data were expressed as perfusion units (PU) calculated using PIMSoft version 1.5 (Perimed). Subsequently, oxygen saturation of the liver and kidney was assessed by tissue spectrophotometry using an “oxygen to see” (O2C) system (LEA Medizintechnik, Giessen, Germany). The O2C probe was placed on the surface of the liver and kidney, and the capillary venous oxygen saturation was recorded.

After these procedures, rats were euthanized and the superior mesenteric arteries (SMAs) were rapidly excised and placed in ice-cold Krebs-Henseleit (K-H) solution. SMAs were sectioned into rings 2-3 mm long, and the endothelium was denuded by gently rubbing the intimal surface as previously described. The vascular contraction function to NE was measured using an isolated organ perfusion system (Scientiﬁc Instruments, Barcelona, Spain). Maximal contraction (Emax) and NE concentration-response curves were used to compare vascular reactivity.

A cohort of twenty-four rats was divided into three groups (n=8/group): shock+LR+Cal 1 mg/kg, shock+LR+Cal+ML-9 10^-6^ mol/L (a selective MLCK inhibitor, Sigma, St. Louis, MO, USA), and shock+LR+Cal+ML-9 5×10^-6^ mol/L. The procedures, including production of the shock model, Cal treatment, and isolation of SMA rings were identical to those described above. Some SMA rings were treated with ML-9 10^-6^ mol/L or 5×10^-6^ mol/L for 30 min before reactivity to NE was determined.

**Microvascular reactivity of mesenteric microvessels *in vivo***

Thirty-two rats were randomly divided into four groups (n=8/group): normal control, shock control, shock+LR, and shock+LR+Cal 1 mg/kg. Two hours after resuscitation, the rats were placed in the lateral position. A 2~3-cm-long midline abdominal incision was made, and the small intestine near the cecum was exteriorized gently and placed on a transparent observation board. The mesenteric microvessels were observed under an inverted intravital microscope (BX51WI, Olympus, Tokyo, Japan) within a thermocontrolled box (37°C). Single unbranched arterioles with diameter between 30 to 50 μm and length ~200 μm were selected for study. The contractile response of mesenteric arterioles was assessed by measuring the change in diameter in response to increasing doses of NE (10^-9^, 10^-8^, 10^-7^, and 10^-6^ mol/L). The videos and images of arterioles were recorded using a microscope equipped with a CCD camera (DP21, Olympus). Arteriole diameters were measured with the Image-Pro Plus 5.0 application (Media Cybernetics, Silver Spring, MD). Changes in diameter were calculated using the expression (D_1_-D_2_)/D_1_×100% (D_1_ and D_2_: diameter before and after the addition of NE, respectively).

**Isolation of cardiomyocytes and VSMCs, and contraction in isolated myocytes**

Thirty-two rats were randomly divided into four groups (n=8/group): normal control, shock control, shock+LR, and shock+LR+Cal 1 mg/kg. Two hours after resuscitation, hearts and mesenteric arteries were rapidly removed. Dissociated cardiomyocytes were isolated by a collagenase perfusion method. Briefly, the hearts were mounted in a Langendorff perfusion apparatus, and perfused with Ca^2+^-free perfusion buffer (mmol/L: NaCl 137, KCl 5.4, MgCl_2_ 1.2, HEPES 10, glucose 10, pH 7.4) containing 10 mmol/L taurine for 7 min, then switched to oxygenated digestion buffer (perfusion buffer containing 2 g/L collagenase type II, 2 g/L bovine serum albumin, and 25 μmol/L CaCl_2_) for 19 min. Hearts were then perfused with perfusion buffer to terminate the digestion. After perfusion, the ventricles were pulled into small pieces in Krebs buffer (KB) solution, and the tissues were gently dispersed to generate a cell suspension using a plastic pipette. Cardiomyocytes were harvested by filtration through a 100-mesh cell strainer, centrifuged at 500 rpm for 40 s, and then washed sequentially with buffers containing, 0.4, 0.8, and 1.2 mmol/L CaCl_2_. Cardiomyocytes contraction was observed using an IonOptix edge-detection system (IonOptix LLC, Milton, MA, USA).

Dissociated smooth muscle cells were freshly isolated using an enzymatic method. Briefly, after dissection and removal of endothelium, the mesenteric arteries were cut into 2 mm segments, and then incubated for 30 min at 37°C with gentle shaking in a Ca^2+^-free physiological salt solution (PSS, mmol/L: NaCl 127, KCl 5.9, MgCl_2_ 1.2, HEPES 10, glucose 12, pH 7.4) containing 2 g/L collagenase type II, 4 g/L papain, 2 g/L bovine serum albumin, and 1 mmol/L dithiothreitol. The partly digested tissues were rinsed three times with Ca^2+^-free PSS and gently agitated with a plastic pipette in low-Ca^2+^ (20 μmol/L) PSS. The dissociated cells were harvested by filtration through a 100-mesh cell strainer, and then stored at 4°C until used (within 4-h following dispersion). The contractile response to NE (measured as change in length) of isolated VSMCs was determined using a Leica TCS SP5 confocal system (Leica Microsystems, Wetzlar, Germany).

**Oxidative stress assay, preparation of tissue lysates, and western blot analysis**

Thirty-two rats were divided into four groups (n=8/group), and treated as described above. Two hours after resuscitation, blood samples and SMA tissues were collected. Serum levels for four oxidative stress biomarkers, including malondialdehyde (MDA), superoxide dismutase (SOD), catalase (CAT), and glutathione (GSH), were detected with commercial assay kits (Jiancheng Bioengineering Institute, Nanjing, China) according to the manufacturer’s instructions. SMA tissue protein extracts were prepared and western blot analysis was performed as described previously. Antibodies were as follows: MLC (myosin light chain, 1:1000; Sigma), phospho-MLC (1:1000; Cell Signaling, Danvers, MA, USA), Drp1 (1:1000; Abcam, Cambridge, MA, USA), Fis1 (1:1000; Abcam), Mfn1 (1:1000; Abcam), Mfn2 (1:1000; Abcam), and β-actin (1:5000; Sigma). Bands were detected with fluorescent secondary antibodies and quantified using the Odyssey CLx Infrared Imaging System (LI-COR, Inc., Lincoln, Nebraska, USA).

**Primary culture of VSMCs and hypoxic treatment**

Rat VSMCs were obtained from the SMAs of twenty adult SD rats using an explant technique as previously described. VSMCs were cultured in Dulbecco’s modified Eagle medium-F12 supplemented with 20% fetal bovine serum (Hyclone, Logan, UT, USA) and 1% antibiotics. Cells from passages 3 to 5 were used in the study. For hypoxic challenge, VSMCs were transferred into a hypoxia culture compartment (MIC-101, Billups-Rothenberg Inc., Del Mar, CA, USA), equilibrated by bubbling with 95% N_2_ and 5% CO_2_ for 15 min and then allowed to sit for 10 min. This procedure was repeated ﬁve times until the oxygen concentration was < 0.2%. VSMCs were maintained under hypoxic conditions for 2 hr and then used for subsequent experiments.

**ROS level in VSMCs**

Reactive oxygen species (ROS) levels in VSMCs were measured using the 2′,7′-dichlorofluorescin diacetate (DCF-DA) method. The cultured VSMCs were divided into three groups: normal control, hypoxia 2-h, and hypoxia+Cal (10 μmol/L, 30 min). After treatment with hypoxia and/or Cal, VSMCs were incubated with 10 μmol/L DCF-DA (Sigma) for 20 min at 37°C. DCF fluorescence was detected at 488 nm excitation and 525 nm emission using a Leica TCS SP5 confocal microscope. Images were collected and the mean fluorescence intensities of DCF were measured using the Image J application.

**Mitochondrial morphology**

To visualize mitochondria in living cells, a mitochondria-specific probe was used. The VSMCs were divided into 3 groups: normal control, hypoxia 2-h, and hypoxia+Cal. After being subjected to hypoxia and/or treated with Cal, the VMSCs were washed with PBS and loaded with 100 nmol/L of MitoTracker deep red (Sigma) for 30 minutes. Fluorescence was detected with an excitation of 633 nm using a Leica confocal microscope.

**Isolation of mitochondria from VSMCs, and western blot analysis**

The isolation of mitochondria from VSMCs were obtained with the Mitochondria Isolation Kit (Sigma). Western blot analysis was performed using antibodies against Drp1 (1:1000; Abcam), Fis1 (1:1000; Abcam), and VDAC1 (used as loading control for mitochondrial proteins, 1:1000; Sigma).

**Statistical analysis**

Data are presented as means ± standard error (SEM). Animal survival was analyzed using the Kaplan-Meier survival curve, and the differences among groups were analyzed using a chi-square test. For the other experiments, differences between experimental groups were assessed using one or two-factor analysis of variance analyses, followed by *post-hoc* Tukey tests. Statistical calculations were performed using SPSS 18.0 (SPSS Inc., Chicago, IL). *P*<0.05 was the threshold for statistical significance.

**2. Supplemental Figures**

**
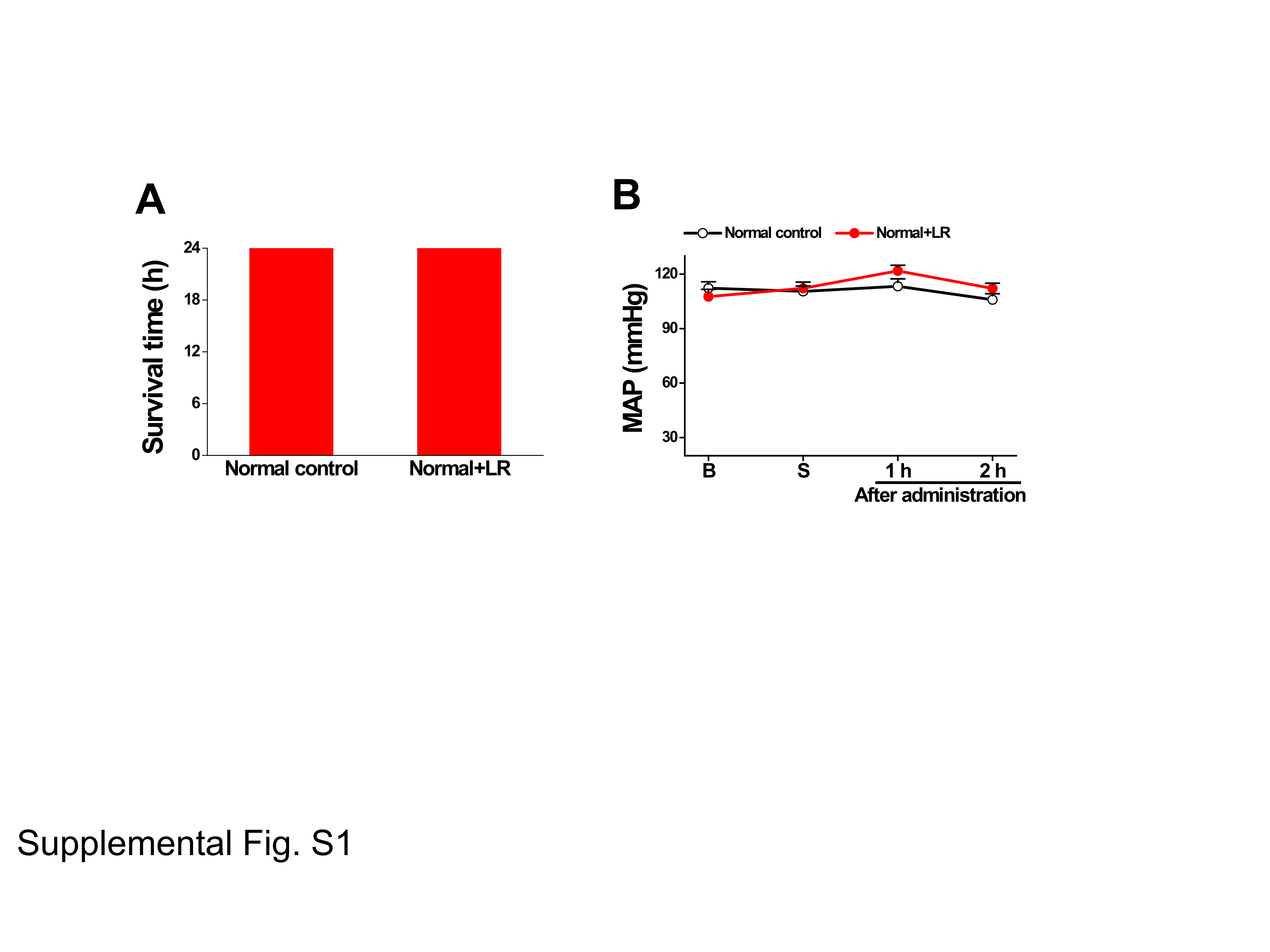
**

**Supplemental Figure S1.** Effect of LR on animal survival and blood pressor in normal rats (n=16/group). A: survival time; B: mean arterial pressure (MAP). **P* <0.05, ***P* <0.01 compared with the normal control group. LR, lactated Ringer’s solution; B, baseline; S, end of shock

**
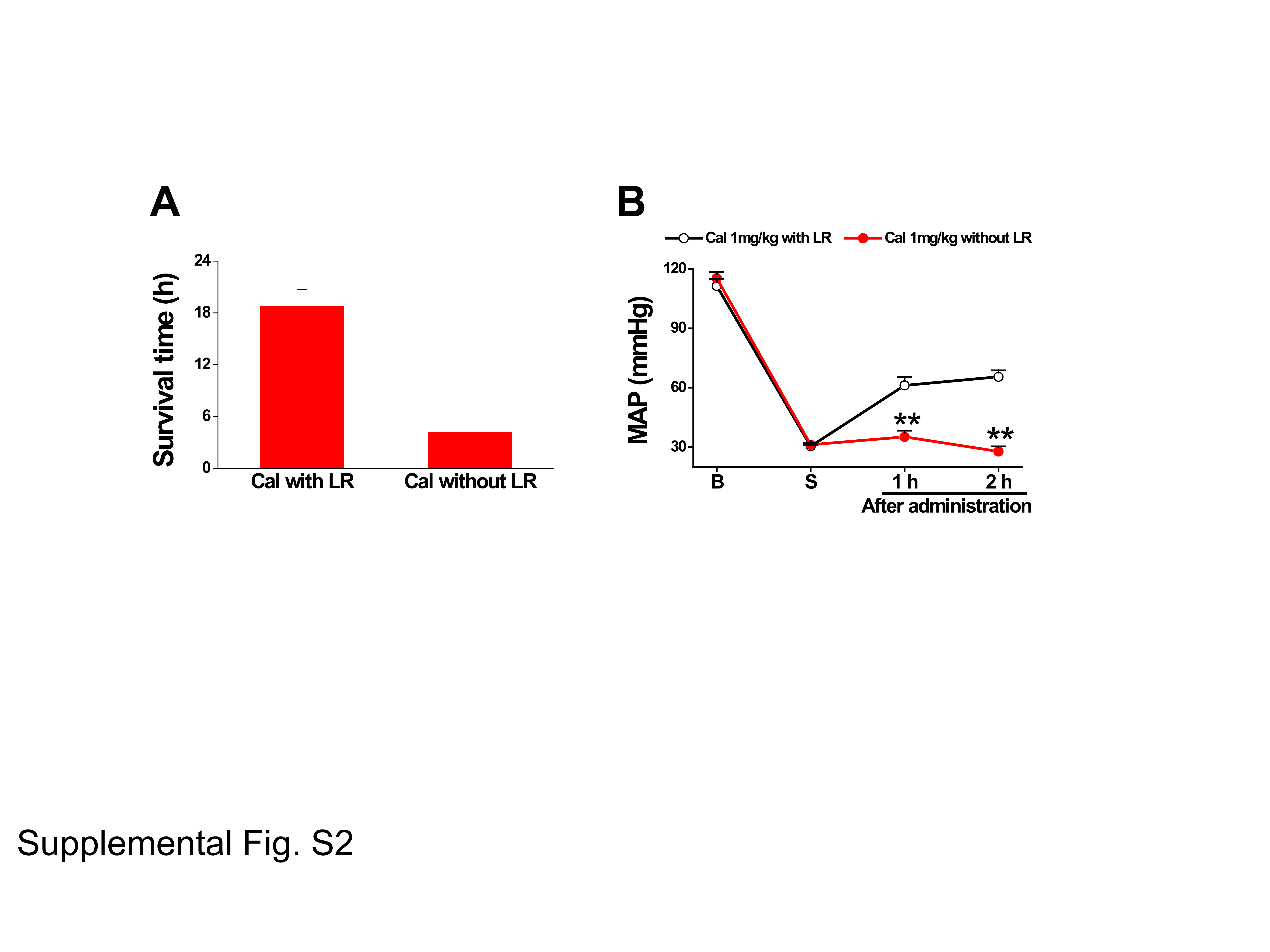
**

**Supplemental Figure S2.** Effect of Cal with or without LR resuscitation on animal survival and blood pressor after traumatic hemorrhagic shock. (n=16/group). A: survival time; B: mean arterial pressure (MAP). **P* <0.05, ***P* <0.01 compared with the Cal with LR group.; LR, lactated Ringer’s solution; Cal, Calhex-231; B, baseline; S, end of shock

**
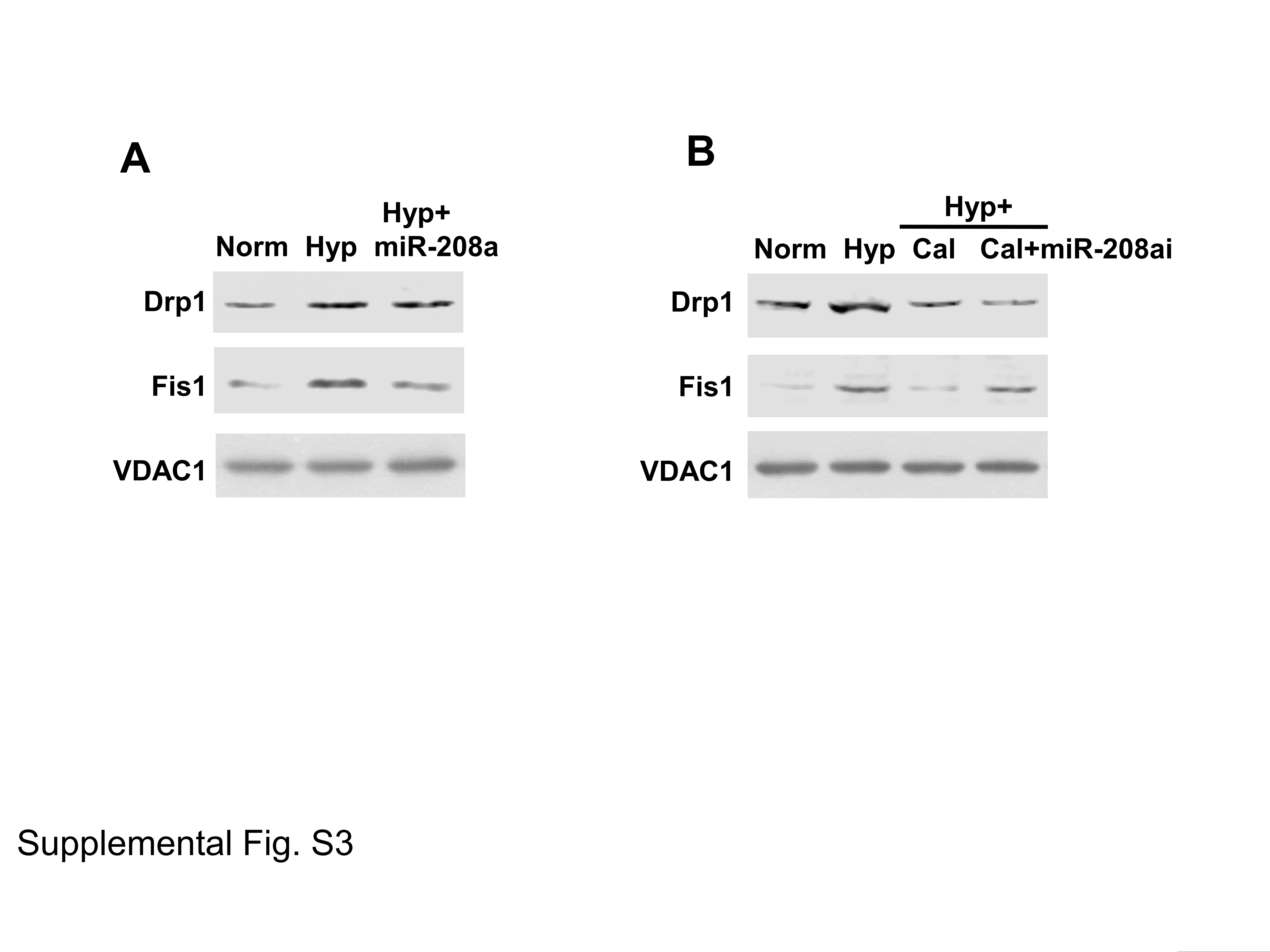
**

**Supplemental Figure S3.** Role of miR-208a in Cal regulating expression of Drp1 and Fis1 in isolated mitochondria from VSMCs. A: effect of miR-208a on the protein expression of Drp1 and Fis1 in in isolated mitochondria from VSMCs. B: role of miR-208a in the regulation of Drp1 and Fis1 expression by Cal in isolated mitochondria from VSMCs. Norm, normal; Hyp, hypoxia; miR-208a, miR-208a mimic; miR-208ai, miR-208a inhibitor; Cal, Calhex-231.


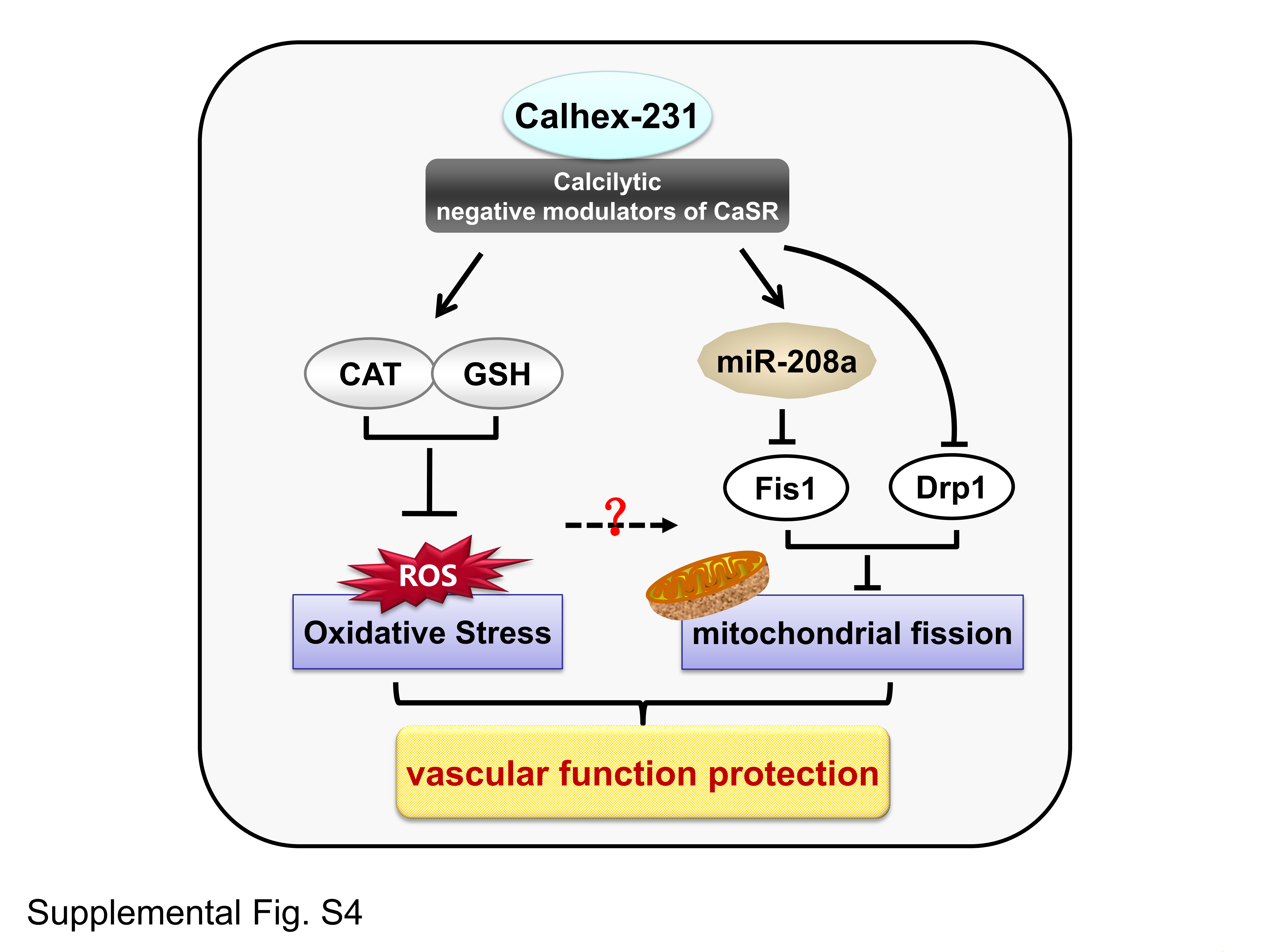


**Supplemental Figure S4.** Schematic diagram showing the possible signaling mechanisms of Calhex-231’s effects under shock states.
